# CD103^+^CD11b^+^ Dendritic Cells Induce T_h_17 T Cells in Muc2-Deficient Mice with Extensively Spread Colitis

**DOI:** 10.1371/journal.pone.0130750

**Published:** 2015-06-29

**Authors:** Ulf A. Wenzel, Caroline Jonstrand, Gunnar C. Hansson, Mary Jo Wick

**Affiliations:** 1 Department of Microbiology and Immunology and the Mucosal Immunobiology and Vaccine Center (MIVAC), Institute of Biomedicine at Sahlgrenska Academy, University of Gothenburg, Gothenburg, Sweden; 2 Department of Medical Biochemistry, Institute of Biomedicine at Sahlgrenska Academy and MIVAC, University of Gothenburg, Gothenburg, Sweden; Charité, Campus Benjamin Franklin, GERMANY

## Abstract

Mucus alterations are a feature of ulcerative colitis (UC) and can drive inflammation by compromising the mucosal barrier to luminal bacteria. The exact pathogenesis of UC remains unclear, but CD4^+^ T cells reacting to commensal antigens appear to contribute to pathology. Given the unique capacity of dendritic cells (DCs) to activate naive T cells, colon DCs may activate pathogenic T cells and contribute to disease. Using Muc2^-/-^ mice, which lack a functional mucus barrier and develop spontaneous colitis, we show that colitic animals have reduced colon CD103^+^CD11b^-^ DCs and increased CD103^-^CD11b^+^ phagocytes. Moreover, changes in colonic DC subsets and distinct cytokine patterns distinguish mice with distally localized colitis from mice with colitis spread proximally. Specifically, mice with proximally spread, but not distally contained, colitis have increased IL-1β, IL-6, IL-17, TNFα, and IFNγ combined with decreased IL-10 in the distal colon. These individuals also have increased numbers of CD103^+^CD11b^+^ DCs in the distal colon. CD103^+^CD11b^+^ DCs isolated from colitic but not noncolitic mice induced robust differentiation of Th17 cells but not Th1 cells *ex vivo*. In contrast, CD103^-^CD11b^+^ DCs from colitic Muc2^-/-^ mice induced Th17 as well as Th1 differentiation. Thus, the local environment influences the capacity of intestinal DC subsets to induce T cell proliferation and differentiation, with CD103^+^CD11b^+^ DCs inducing IL-17-producing T cells being a key feature of extensively spread colitis.

## Introduction

The intestinal environment poses a challenge to the immune system by requiring tolerance to luminal antigens yet must maintain the capacity to respond to pathogen encounter. Disruption of this balance can lead to inflammatory disorders such as inflammatory bowel disease. Central to this balancing act are intestinal mononuclear phagocytes (iMPs) such as dendritic cells and macrophages (MΦ) [1–5]. The exact role of these cell types in intestinal homeostasis is not fully understood, but during steady state, iMPs have anti-inflammatory properties that, in the case of MΦs, depends on IL-10 signaling [6–8]. During induced inflammation the differentiation of monocyte-derived iMPs is altered and cells with pro-inflammatory properties accumulate [9–12].

Intestinal DCs, particularly those expressing the integrin CD103, are of specific interest as they continuously migrate from the intestine to the mesenteric lymph nodes (MLN) to initiate T cell responses [3,13–15]. Functionally, DCs expressing CD103 have a superior capacity to induce T cells to home to the intestine and under steady state can drive the generation of Tregs via the vitamin A metabolite retinoic acid [15]. Use of CD103 combined with CD11b have revealed distinct subsets of iMPs that differ in origin and function [2,3,9–11,14–17]. Among these iMPs, three DC populations migrate from the intestinal lamina propria (LP) to the MLN in steady state (CD103^+^CD11b^-^ DCs, CD103^+^CD11b^+^ DCs and CD103^-^ DCs) and induce proliferation and differentiation of T cells [3,14]. CD103^+^CD11b^-^ DCs are unique in their capacity to cross-present intestinal antigens to prime CD8 T cell responses in vivo [3] while CD103^+^CD11b^+^ DCs are important for intestinal Th17 cell differentiation through non-cognate interactions [17–19]. Whether a particular intestinal DC subset is predominantly responsible for inducing Tregs *in vivo* is not clear. Indeed, evidence thus far suggests functional redundancy between CD103^+^CD11b^-^ DCs and developmentally independent CD103^+^CD11b^+^ DCs in maintaining intestinal Treg numbers [19]. Although CD103^+^CD11b^-^ DCs can induce IFNγ production [2], neither these DCs nor CD103^+^CD11b^+^ DCs are required for induction of IFNγ-producing CD4^+^ T cells [17–19]. iMPs also contain a heterogeneous population of CD103^-^ cells that includes bona fide DCs that are distinct from macrophages [2,14]. CD103^-^ DCs are a minor population migrating from the intestine, tend to produce pro-inflammatory cytokines, promote Th1 or Th17 cells, and exacerbate colitis in transfer models [2,14].

Despite increased insight into the function of iMPs during inflammation, most information has been gained from chemically induced or adoptive T cell transfer models. Here we use Muc2^-/-^ mice that lack protective Muc2 mucin in the colon and develop spontaneous colitis with features resembling human ulcerative colitis [20–23]. The spontaneous nature of the Muc2^-/-^ model provides the advantage of examining mice at different stages of disease in the absence of chemically induced, bolus disease induction. We reveal distinct changes in iMP populations and cytokine patterns that distinguish colitis localized distally from extensively spread colitis. We also show that the different DC populations from colitic versus noncolitic environments have distinct capacities to induce CD4 T cell proliferation and differentiation. The Muc2^-/-^ spontaneous colitis model gives insights into how colitis affects iMP function and subsequent T cell responses, which increases our understanding of intestinal homeostasis and its disruption in inflammatory bowel disease.

## Results

### Colitis spreads proximally from the rectum in the absence of Muc2

**Fig 1:**
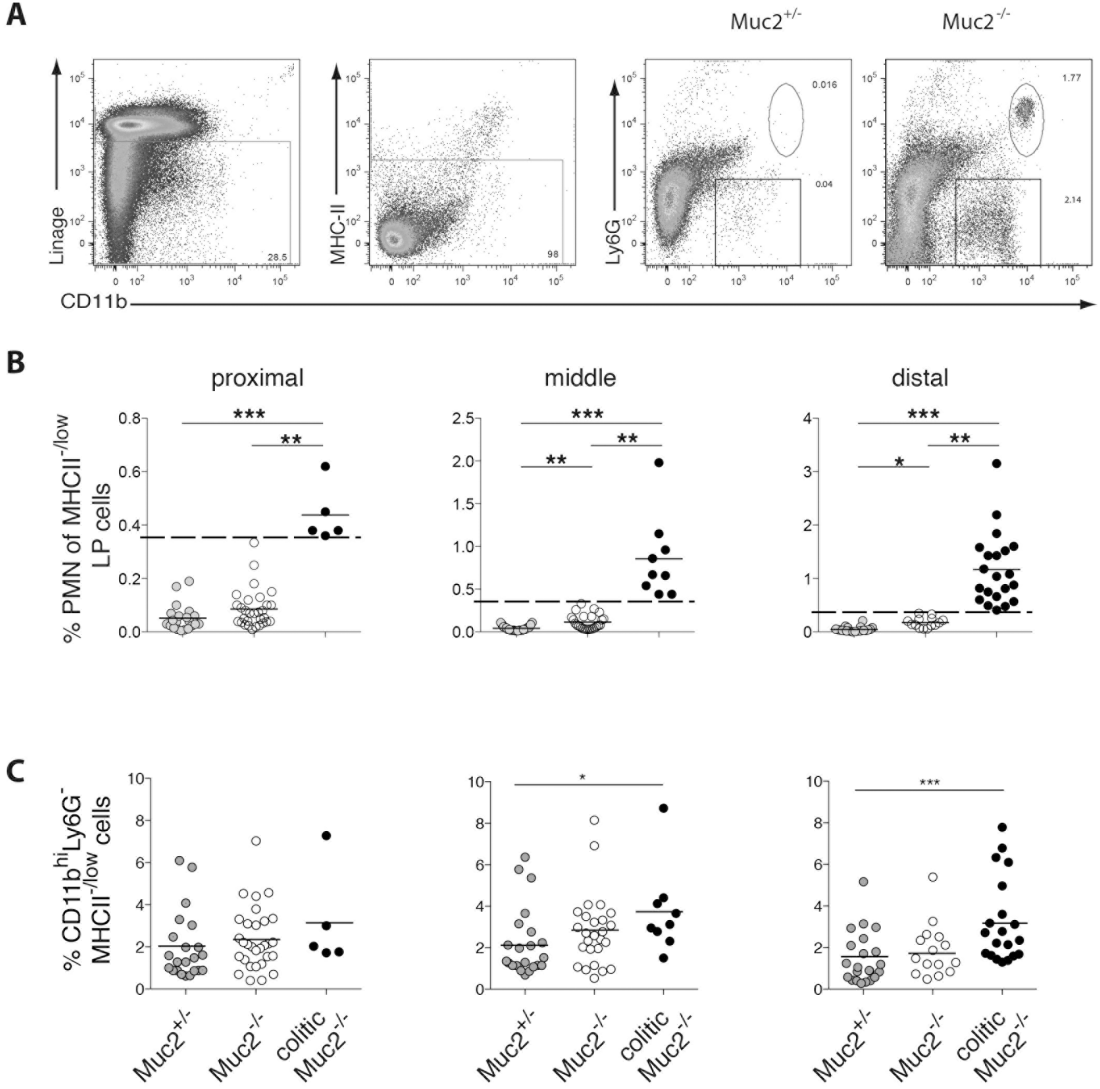
Local accumulation of neutrophils in the colon of Muc2^-/-^ mice. LP cells from proximal, middle or distal colon sections of Muc2^-/-^ and Muc2^+/-^ littermates were analyzed by flow cytometry. (A) Viable neutrophils were identified as 7AAD^-^MHCII^-^CD11b^+^Ly6G^+^ cells. The numbers represent the percent of cells in the indicated gate. (B) The frequency of neutrophils among viable LP cells for all mice examined is shown. The dashed line indicates the “cut off” value of 0.36%[23]). (C) The frequency of CD11b^+^MHC^-/low^ cells among viable LP cells of all mice examined is depicted. Each symbol represents an individual mouse and the horizontal line indicates the mean. Results are from 12 independent experiments with a total of 14–22 mice per group. Statistical significance was assessed using the Kruskal-Wallis test followed by Dunn’s multiple comparison test.

Recently, we demonstrated that spontaneous intestinal inflammation in Muc2^-/-^ mice is restricted to the colon and shares features of human ulcerative colitis [23]. In this study, Muc2^-/-^ mice were placed into colitic versus non-colitic groups based on neutrophil influx in the whole colon, using a threshold of > 0.36% neutrophils among viable LP cells to define colitic mice [23]. Because human ulcerative colitis proceeds proximally from rectum to cecum, we hypothesized that infiltration of neutrophils in Muc2^-/-^ mice would be more prominent in the distal than the proximal colon. Hence, we analyzed neutrophil infiltration into the LP of proximal, middle and distal colon segments of Muc2^+/-^ and Muc2^-/-^ mice. This revealed that 5 of 35 Muc2^-/-^ mice had > 0.36% neutrophil in the proximal colon LP and were thus classified as colitic in this colon segment (Fig 1A and 1B). In the distal colon, however, these 5 mice plus 16 additional Muc2^-/-^ mice, but no Muc2^+/-^ mice, had > 0.36% neutrophils (Fig 1A and 1B). No change in neutrophil frequency was apparent among small intestinal LP cells regardless of the neutrophil frequency in any of the colon segments of the same individual or age of the mice (data not shown and [23]). In addition, CD11b^hi^MHCII^-/low^ cells were increased in mice classified as colitic based on increased neutrophils, particularly in the distal colon (Fig 1A and 1C). Thus, spontaneous colitis in Muc2^-/-^ mice monitored by neutrophil infiltration is most frequently observed in the distal colon and correlates with the influx of CD11b^hi^MHCII^-/low^ cells.

### Reduced CD103^+^CD11b^-^ DCs and increased CD103^-^CD11b^+^ iMPs characterize colitis in Muc2^-/-^ mice

**Fig 2:**
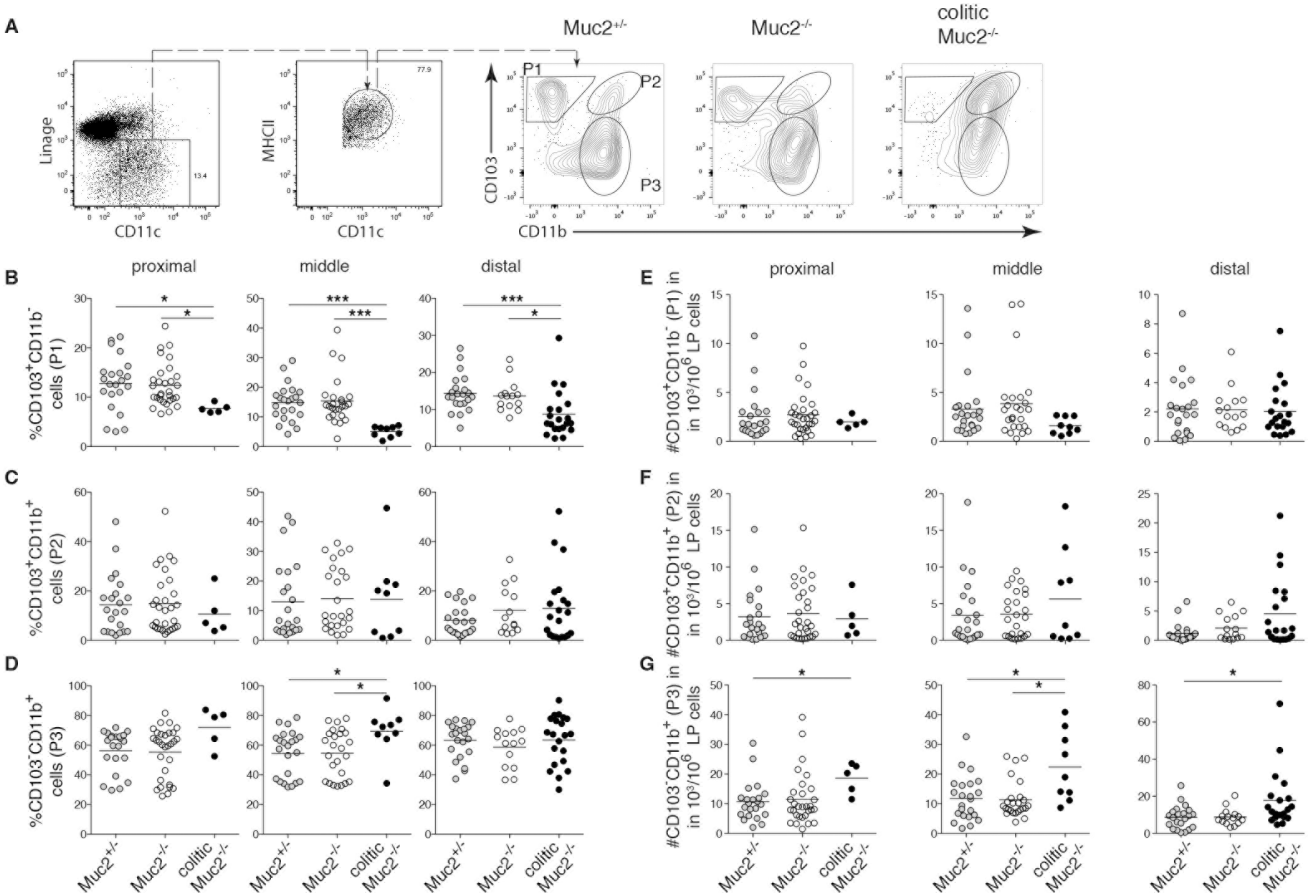
Altered frequency of iMP populations in the colon of Muc2^-/-^ mice. LP cells from proximal, middle and distal colon of Muc2^+/-^ controls, Muc2^-/-^, and colitic Muc2^-/-^ mice were analyzed by flow cytometry. (A) A representative dot plot of a Muc2^+/-^ mouse showing gating of viable NK1.1^-^TCR^-^CD19^-^CD11c^+^MHC-II^+^ cells is depicted in the left two dot plots and further analysis of gated CD11c^+^MHC-II^+^ cells for expression of CD103 and CD11b for the indicated genotype of mouse is shown to the right. B-D depict the frequency of CD103^+^CD11b^-^ "P1" DCs (B), CD103^+^CD11b^+^ "P2" DCs (C), and CD103^-^CD11b^+^ "P3" iMPs (D) among viable LP cells in the indicated gates. E-G show the absolute number of viable CD103^+^CD11b^-^ "P1" DCs (E), CD103^+^CD11b^+^ “P2” DCs (F) and CD103^-^CD11b^+^ “P3” iMPs (G). Each symbol represents an individual mouse. For B-G results from the proximal (left column), middle (center column) and distal (right column) colon are shown. The mean of each group is indicated by the horizontal line. Segregating Muc2^-/-^ mice into colitic and non-colitic is performed according to neutrophil influx in the respective colon segment. Data are from 12 independent experiments that analyzed 14–22 mice per group. Statistical significance between groups was assessed using the Kruskal-Wallis test followed by Dunn’s multiple comparison test.

We hypothesized that the local composition of iMPs, particularly those identified by CD103 and CD11b, could influence local inflammation. Therefore, the frequency of CD103^+^CD11b^-^ (P1) and CD103^+^CD11b^+^ (P2) DC subsets in the colon LP was determined. In addition, the frequency of CD103^-^CD11b^+^ (P3) iMPs, which contains both conventional DCs and CD64^+^ monocyte-derived cells [2,9–11,14,24,25], was assessed. Analyzing the three colon segments separately showed that the percent of P1 DCs among MHCII^+^CD11c^+^ cells was reduced in all colon segments in colitic mice while their absolute number did not change (Fig 2A,2B and 2E). Neither the frequency nor number of P2 DCs was significantly altered in colon segments of colitic Muc2^-/-^ mice (Fig 2A,2C and 2F). In contrast, the number of P3 iMPs increased in all colon segments (Fig 2A,2D and 2G). Thus, the spontaneous inflammation characterized by neutrophil infiltration in the colon LP of Muc2^-/-^ mice corresponded with decreased frequency of P1 DCs and an increased number of P3 iMPs.

### Inflammatory cytokines characterize the distal colon of colitic Muc2^-/-^ mice

**Fig 3:**
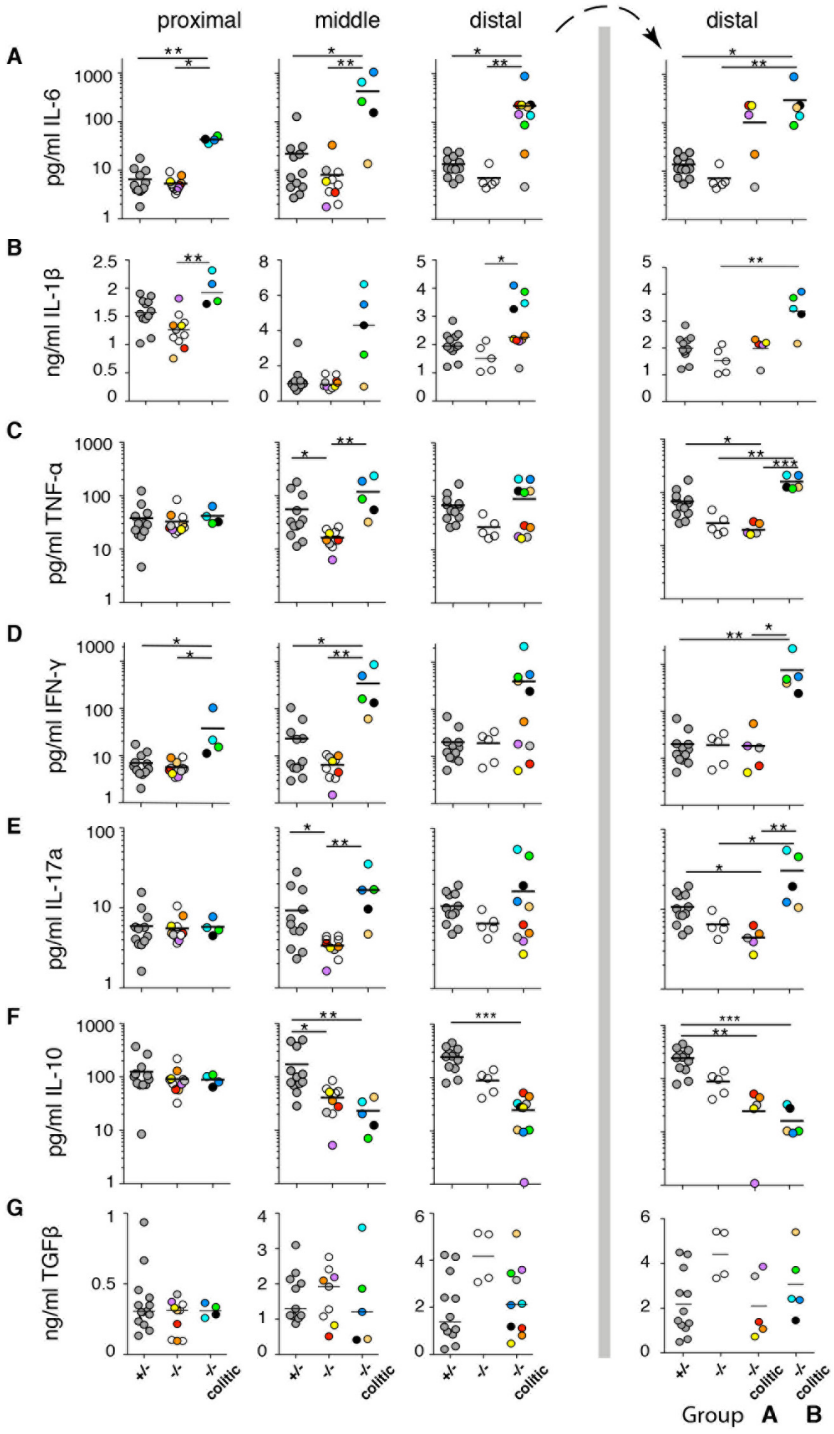
Inflammatory cytokines characterize the distal colon of a subgroup of colitic Muc2^-/-^ mice. Saponin extracts of proximal, middle and distal colon were prepared and normalized to total protein concentration. Cytokine expression was analyzed by CBA (A, C- F) or ELISA (B, G). Each symbol represents an individual mouse and the solid line indicates the mean of each group. Symbols of the same color in Figs 3 and 4 and S1 Fig represent samples from the same colon segment of the same animal. Mice are segregated into colitic and non-colitic according to neutrophil influx into the colon LP in the respective colon segment. Data are pooled from 5 independent experiments that examined 27 mice. The distal colon panels to the right of the vertical bar show the same distal colon data as to the left of the bar except that the colitic mouse group is segregated into group “A” and “B”. Statistical significance between groups was assessed using the Kruskal-Wallis test followed by Dunn’s multiple comparison test.

To better understand the nature of the inflammation in Muc2^-/-^ mice, we assessed the local cytokine environment and correlated it with the local cellular composition in the same colon segment of the same individual. To achieve this, the proximal, middle and distal colon segments were each divided in half and cytokines were quantified in one half, while cell populations were analyzed by flow cytometry in the remaining half. In general, inflammatory cytokine production increased from the proximal to distal colon (Fig 3A–3E), which was particularly apparent for IL-6. In the distal colon of colitic mice, the concentration of pro-inflammatory cytokines seemed to segregate the mice into subgroups (Fig 3, far right, groups “A” and “B”). That is, group B colitic mice had higher levels of IL-6, IL-1β, TNFα, IFN-γ and IL-17a together with significantly lower IL-10 in the distal colon. These same mice were regarded as colitic in all three sections of the colon except for one mouse (beige symbol), which did not have elevated neutrophils in the proximal colon (Figure A in S1 Fig). In contrast, group A colitic mice had either unchanged or reduced levels of the pro-inflammatory cytokines in the distal colon as well as reduced IL-10 (Fig 3A–3G, far right). Unlike group B mice, group A animals had increased neutrophil influx only in the distal colon (Figure A in S1 Fig and Fig 3). Thus, colitic mice can be divided into A and B subgroups based on cytokine profile in the distal colon and whether increased neutrophils has spread proximally.

In contrast to the other cytokines, TGF-β did not reveal any clear patterns or clustering of mice and was not correlated with inflammation *per se*, although non-colitic Muc2^-/-^ mice had a trend of elevated TGF-β in the distal colon (Fig 3G; open symbols). Moreover, only minor amounts of IL-2 and no IL-4 above background was detected (data not shown). Overall, cytokine profiles revealed two clusters of colitic mice, and individuals falling into group B had colitis spread from the distal to the proximal colon.

### Increased CD103^+^CD11b^+^ (P2) DCs characterizes the distal colon of group B colitic mice

**Fig 4:**
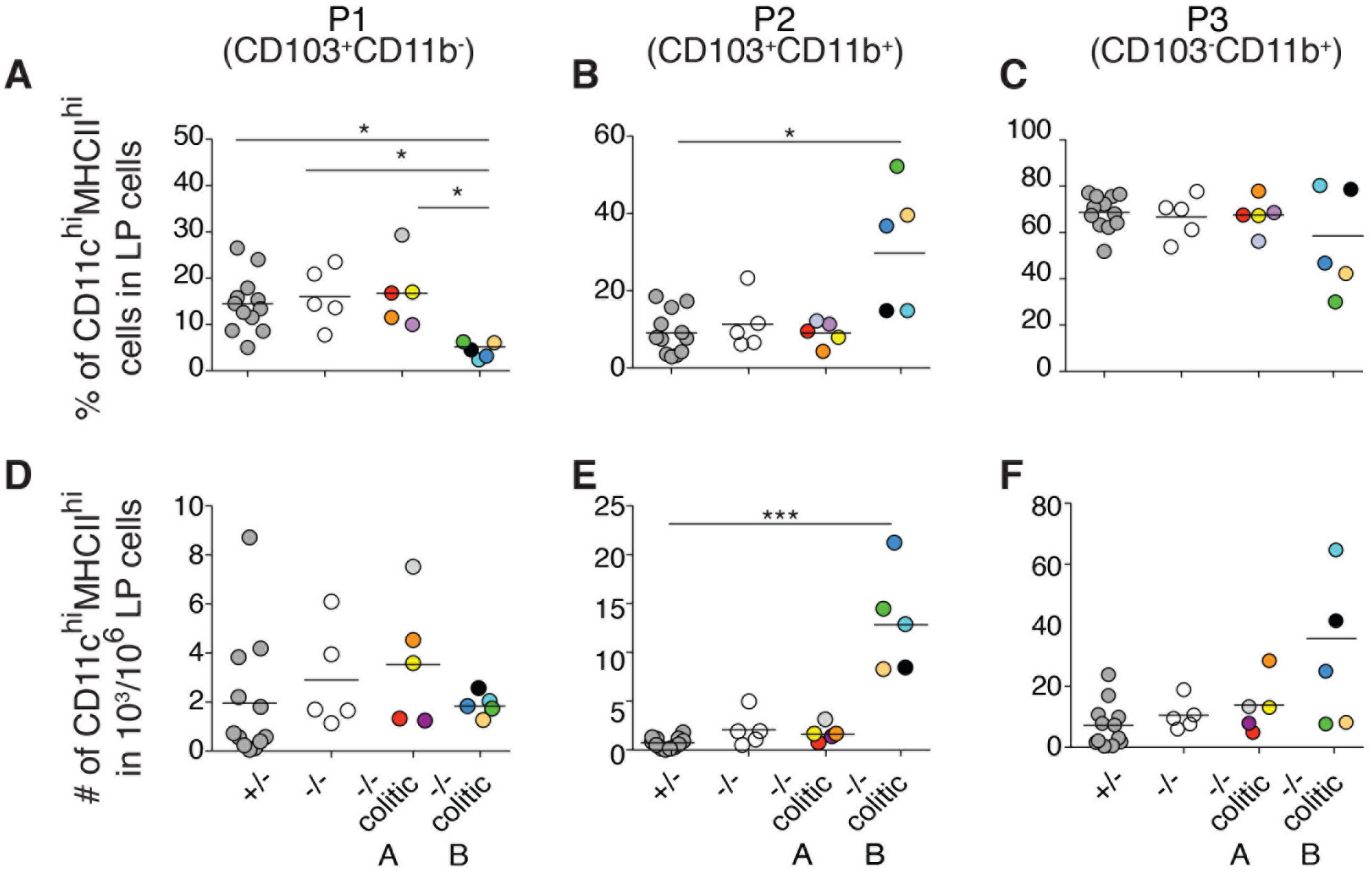
Cytokine profiles in the distal colon of colitic mice correlate with increased CD103^+^CD11b^+^ P2 DCs in the same colon segment. LP cells of the other half of the same distal colon segment of the same mice used for cytokine analyses in Fig 3 were analyzed by flow cytometry. Cells were gated as in Fig 2A. (A-C) The frequency of DCs belonging to subsets P1 (A), P2 (B) or P3 (C) among viable MHCII^+^CD11c^hi^ cells is shown. (D-E) The absolute number of DCs belonging to subsets P1 (D), P2 (E) or P3 (F) among 10^6^ viable LP cells is depicted. Each symbol represents an individual mouse and the solid line indicates the mean of each group. Statistical significance between groups was assessed using the Kruskal-Wallis test followed by Dunn’s multiple comparison test.

Next, we correlated the cytokine profiles in the distal colon of the A and B subgroups of the colitic mice in Fig 3 with the iMP balance quantitated in the other half of the same distal colon segment. This revealed a reduced frequency of P1 DCs (Fig 4A and 4D) and concomitant increase in the frequency and number of P2 DCs in group B mice (Fig 4B and 4E). However, a correlation between P3 iMPs with cytokine profiles was not observed (Fig 4C and 4F). Thus, group B colitic mice, which had a distinct and homogenous cytokine profile in the distal colon (Fig 3, far right), had a heterogeneous number of P3 cells in this same colon segment that was not significantly increased (Fig 4C and 4F). Instead, group B colitic mice had increased P2 DCs in the distal colon (Fig 4B and 4E).

Thus, although both A and B colitic mice have neutrophil influx in the distal colon, group B has elevated pro-inflammatory cytokines and increased P2 DCs in the distal colon, while group A does not. Importantly, influx of neutrophils in group B mice extends to the proximal and middle colon while in group A mice it remains confined to the distal colon. This suggests that group A mice have colitis localized distally while group B animals have extensive colitis that has spread proximally.

The chemokines CXCL1 and CXCL2 attract neutrophils during inflammation while CCL9 is a chemoattractant for CD11b^+^ intestinal DCs[26,27]. To investigate the chemokines directing neutrophil and P2/P3 DC recruitment into the colon segments, we performed quantitative real time PCR (qPCR) on total cell suspensions from the same colon segments (proximal, middle or distal) from the same animals analyzed in Figs 3 and 4 and Figure A in S1 Fig. Expression of CXCL1 in the distal colon of Muc2^-/-^ colitic group B mice was significantly increased relative to Muc2^-/-^ group A colitic mice (Figure B in S1 Fig). CXCL2 expression in the distal colon of group B mice tended to be greater than that of group A mice, although not statistically significant, but was significantly increased relative to noncolitic Muc2^-/-^ mice (Figure B in S1 Fig). Thus, increased expression of CXCL1, and to a lesser degree CXCL2, characterized the distal colon of group B but not group A colitic mice. In contrast, differences in CCL9 expression between any of the Muc2^-/-^ mouse groups were not apparent.

### The local environment influences the capacity of iMPs to induce T cell proliferation and differentiation

**Fig 5:**
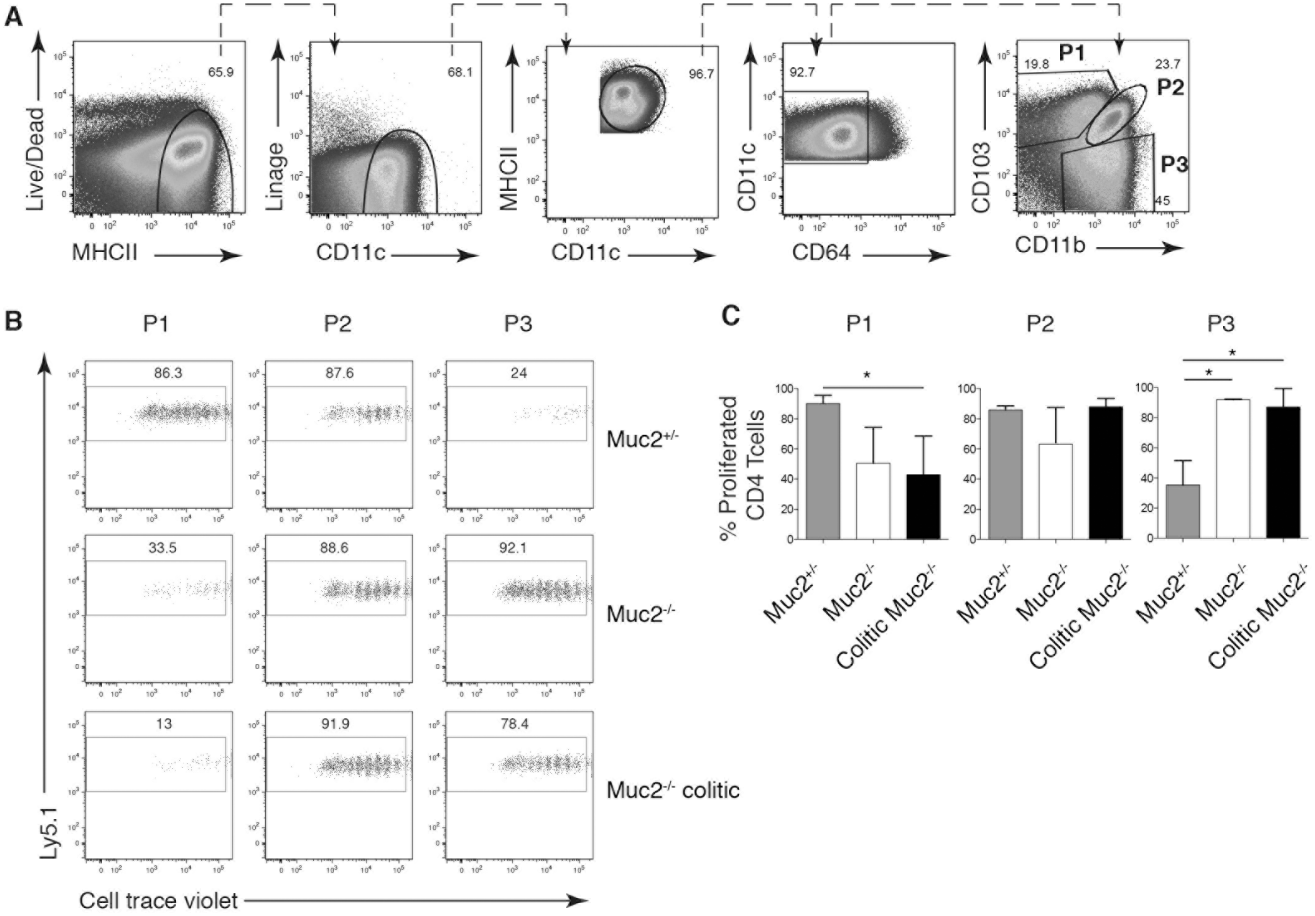
Colitis influences the ability of intestinal DC subsets to induce CD4^+^ T cell proliferation. P1, P2 and P3 cells were sorted from pooled MLN from Muc2^+/-^, Muc2^-/-^ or colitic Muc2^-/-^ and analyzed by flow cytometry. Sorted cells were pulsed with OVA_(323–339)_ peptide prior to co-incubation with CTV-labeled OT-II cells for 5 days. (A) The gating strategy used to identify P1, P2, and P3 populations from MLN cell suspensions is shown. (B) Dot plots show CTV dilution of OT-II cells co-cultured with the indicated iMP subset pulsed with OVA_(323–339)_ peptide. (C) Bar graphs show the frequency of proliferating OT-II T cells ± SD from (B).

Finally, we wanted to investigate the capacity of P1, P2 and CD64^-^ P3 DCs purified from colitic and non-colitic mice to influence proliferation and cytokine production by CD4^+^ OT-II T cells. The paucity of colon LP DCs, coupled with the difficulty obtaining large numbers of mice that have colitis on the same day, hindered isolating sufficient numbers of sorted DC subsets to perform functional experiments. Moreover, analyzing the function of DCs from the lymph nodes draining the colon would give insight into the function of DCs at the sites of T cell priming. We thus tried to isolate DCs from the colon-draining caudal lymph node [28]. We could identify the same three DC subsets in the caudal lymph node (S2 Fig), but again in not high enough numbers to perform functional analyses. Next, given the higher bacterial burden in MLN of Muc2^-/-^ mice [23], we reasoned that the MLN, at least in part, are involved in the detected immune response. Using an antibody cocktail to exclude monocytes (Ly6C) and macrophages (F4/80 and CD64) the same DC subsets identified by CD103 and CD11b were present in the colon LP, caudal lymph nodes and MLN (S2 Fig). Thus, for subsequent functional studies, MLN DCs gated to exclude CD64^+^ macrophages [2,3,11] were used for subsequent co-incubation studies. Hence, the colitis state was determined by quantitating neutrophils in the distal colon and the DC subsets were sorted from MLN (Fig 5A). Co-culture of OT-II cells with purified DCs pulsed with Ova_323-339_ peptide revealed that P1 DCs from control Muc2^+/-^ MLNs are potent inducers of T cell proliferation while the same cells purified from MLNs of Muc2^-/-^ and colitic Muc2^-/-^ mice had a poor capacity to induce OT-II proliferation (Fig 5B and 5C). In contrast, P3 DCs from Muc2^-/-^ and colitic Muc2^-/-^ MLNs, but from not control Muc2^+/-^ MLNs, induced extensive proliferation (Fig 5B and 5C). P2 DCs purified from all three groups of mice induced similar OT-II proliferation (Fig 5B and 5C).

**Fig 6:**
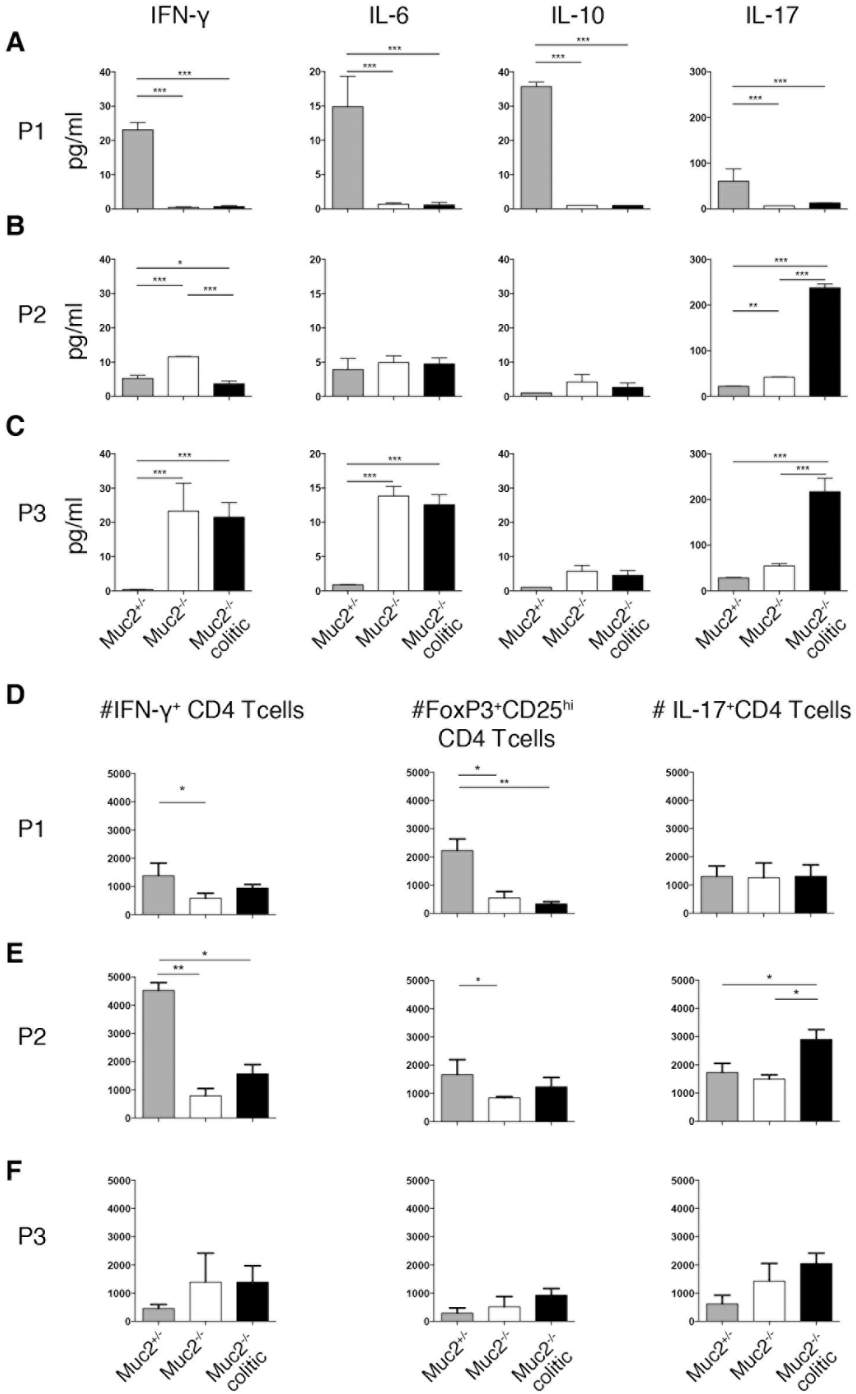
Colitis influences CD4 T cell differentiation induced by intestinal DC subsets. Cytokine concentrations in the supernatants of the T cell proliferation assays in Fig 5 were analyzed by Luminex and are shown in (A-C). (D-F) show the absolute number of differentiated CD4 T cells identified by staining for intracellular IFN-γ (left), CD25 and intracellular FoxP3 (middle) or intracellular IL-17A (right). Data are normalized to 10^4^ CD4 T cells in co-cultures with the indicated DC subset after 5 days. Pooled data from 2 independent experiments in duplicates with a total of 6–8 animals/group is shown ± SD. Statistical significance between groups was assessed using the Kruskal-Wallis test followed by Dunn’s multiple comparison test.

Consistent with the robust proliferation induced by P1 DCs from Muc2^+/-^ MLNs, significantly more IFN-γ, IL-6, IL-17 and IL-10 accumulated in these supernatants compared to P1 DCs from Muc2^-/-^ MLNs (Fig 6A). Co-cultures of P1 DCs from Muc2^+/-^ MLNs also had increased numbers of IFN-γ^+^ and CD25^+^FoxP3^+^ CD4 T cells (Fig 6D and data not shown). P3 DCs from Muc2^+/-^ MLNs, however, induced neither cytokines in co-culture supernatants nor increased number of Th cell subsets (Fig 6C and 6F). In contrast, the robust proliferation of OT-II cells co-cultured with P3 DCs from MLNs of Muc2^-/-^ and colitic Muc2^-/-^ mice was reflected in significantly more IFN-γ, IL-6 and IL-17 in culture supernatants (Fig 6C) while significantly increased numbers of Th1 or Th17 cells were not apparent despite a trend to increased numbers (Fig 6F). P2 DCs from colitic Muc2^-/-^ mice induced more IL-17, which was reflected in the number of differentiated CD4 T cells identified by intracellular cytokine staining (Fig 6E). In contrast, P2 DCs from non-colitic Muc2^-/-^ mice induced more IFN-γ. Differences in viability between P1, P2 and P3 cells in the cultures at the time of analysis did not account for differences in proliferation or cytokine production (data not shown). Thus, T cell proliferation, differentiation and cytokine production is influence by the inflammatory status of the intestine and the iMP population that presents the antigen.

## Discussion

We found that neutrophil influx in the proximal and middle colon was only detected in Muc2^-/-^ mice that also had increased neutrophils in distal colon. This was mirrored by increases in CD103^-^ (P3) iMPs and indicates that these mice have more extensive colitis than animals with increases in the distal colon only. The data suggest that spontaneous colitis in Muc2^-/-^ mice is progressive, starting distally and extending towards the caecum, which mimics human ulcerative colitis. While neutrophils may perpetuate colitis by releasing cytotoxic products and inflammatory mediators, P3 iMPs could contribute by producing pro-inflammatory cytokines [9–12] and/or by driving Th1 or Th17 differentiation [2,11,14]. Observations that P3 iMPs can up-regulate CCR7 suggest that they could contribute to colitis by initiating a harmful T cell response in draining lymph nodes and/or stimulating effector T cells in the LP [2,3,9,14,24]. Induced colitis models also show changes iMP composition analyzed in the whole colon, with an increase in inflammatory monocyte/macrophage iMPs relative to DCs [9–12].

Analysis of multiple immune parameters in different colon segments of the same animal revealed the striking finding that the cytokine pattern in the distal colon of colitic Muc2^-/-^ mice segregated individuals into two subgroups: one with significantly elevated pro-inflammatory cytokines (group B) and one without (group A). Group B mice also had neutrophil influx and increased pro-inflammatory cytokines in at least one additional colon segment while group A did not. These features of group B mice suggest they have extensive colitis that has spread proximally, while group A animals has colitis confined distally. Intriguingly, all mice with extensive colitis had significantly increased P2 (CD103^+^CD11b^+^) DCs while some also had increased P3 iMPs. However, a correlation between the heterogeneous P3 population [2,14] and extensive colitis could not be found. Increased P3 iMPs may thus be a more general indicator of inflammation and be a feature of mice with local colitis, while increased P2 DCs occurs only in mice with colitis spread proximally. Consistent with increased P2 iMPs in the distal colon of group B colitic mice, expression of CCL9, which was shown to attract CD11b^+^ DCs in the small intestine [26], was also increased in the distal colon of these animals. However, the increase was significant relative to Muc2^+/-^ mice but not to group A colitic mice. Thus, differential CCL9 expression in the distal colon of group B colitic mice is not sufficient to explain the differential influx of P2 iMPs relative to group A colitic mice. It is likely that a complex chemokine environment mediates differential attraction of myeloid cell populations into different colon segments during colitis. The neutrophil chemoattractant CXCL1 was increased in group B relative to group A colitic mice in the distal colon but not the proximal or middle colon segments. Additional mechanisms, such as post transcriptional regulation or other factors that influence neutrophil influx in the proximal and middle colon segments of group B mice, may contribute to the complexity of neutrophil attraction during colitis [27,29].

Functionally, P2 DCs are important for intestinal Th17 cell development in a mechanism partially dependent on IL-6 but independent of cognate interactions[17–19]. Moreover, it was demonstrated that P2 DCs induce Th17 cell proliferation during DSS colitis [30]. Consistent with this, Muc2^-/-^ mice with extensive colitis had increased IL-17 in the distal and middle colon. They also had increased IL-6 and IL-1β, which are implicated in Th17 development [31,32], in more than one colon segment. Although TGFβ can influence development of Th17 and T regs depending on the local cytokine environment [31–33], we could not correlate TGFβ levels with colitis status. Interestingly, noncolitic Muc2^-/-^ mice had a trend towards increased TGFβ in the distal colon. This, combined with high IL-10 in the distal colon, could indicate that noncolitic Muc2^-/-^ mice were keeping inflammation at bay through the anti-inflammatory functions of IL-10 and TGFβ on immune cells. Alternatively, IL-10 also seems to have effects on the regulation of mucus production and properties. For example, mice lacking IL-10 have a colon mucus layer that, despite normal appearance, allows bacteria and bacteria-sized beads to penetrate and reach the epithelium causing inflammation [21]. Thus, IL-10 could also promote tolerance through effects on mucus integrity. In contrast, the significantly reduced IL-10 in both A and B groups of colitic Muc2^-/-^ mice may allow the pro-inflammatory cytokines to prevail [34] and lead to pathology.

P1 DCs from Muc2^+/-^ controls, but not from Muc2^-/-^ littermates, were potent inducers of OT-II proliferation and supernatants from cocultures with Muc2^+/-^ P1 DCs contained cytokines with pro-inflammatory capacity. However, these supernatants were also the only ones with significant IL-10, and P1 DCs from Muc2^+/-^ mice induced FoxP3^+^ T cells significantly better than P1 DCs from Muc2^-/-^ mice. This suggests that P1 DCs from the colon of mice with an intact mucus layer are capable of promoting a tolerogenic environment, perhaps through the effects of IL-10 on immune cells and/or the mucus layer or through the ability of Muc2 to impart anti-inflammatory properties on DCs in a β-catenin-dependent fashion [35,36]. Moreover, this tolerogenic-promoting property dominates *in vivo* even though T cells removed from the *in vivo* environment and co-cultured with Muc2^+/-^ P1 DCs could produce pro-inflammatory cytokines. In contrast, the intestinal environment in Muc2^-/-^ mice seems to negatively impact the ability of P1 DCs to induce OT-II proliferation regardless of ongoing colitis.

In contrast to P1 DCs, P3 DCs from Muc2^-/-^, but not Muc2^+/-^ mice, induced robust OT-II proliferation and co-culture supernatants contained IFNγ and IL-17, consistent with higher inflammatory potential of LP DCs from Muc2^-/-^ mice that varies somewhat among DC subsets[14]. Interestingly, IL-17 was the only cytokine produced *ex vivo* by both P3 and P2 DCs from colitic but not non-colitic Muc2^-/-^ mice. Moreover, increased P3 iMPs or P2 DCs *in vivo* in mice with local or severe colitis, respectively, and their IL-17 production *ex vivo*, suggests a role for IL-17 in spontaneous colitis in Muc2-deficient mice, particularly group B mice with extensively spread colitis, and mimics the situation in human ulcerative colitis[37,38]. Moreover, the data suggest that P3 iMPs have a role in initial colitis confined distally while P2 DCs play a role in driving colitis in Muc2^-/-^ mice that spreads proximally.

Our data suggest that the inflammatory status of the colon impacts the function of iMPs. That the microenvironment impacts the function of specific cell types has been studied in tumor biology and is underscored by the shift of tumor-associated macrophages from pro-inflammatory type-1 to tolerogenic type-2 over time [39–41]. An impact of the colitic environment on iMP function *in vivo* is supported by studies showing differential cytokine production in steady-state iMPs cultured in the presence or absence of microbial stimuli [2,9,12,14]. Indeed, changes in the local cytokine environment in the colon, such as that induced by commensal bacterial penetration due to a defective mucus barrier in Muc2^-/-^ mice, influences iMP function and tips the homeostatic balance towards colitis. Understanding the role of defined iMP populations in spontaneous colitis will provide insight into new treatment strategies for inflammatory bowel disease.

## Materials and Methods

### Mice

Muc2^-/-^ mice on the C57BL/6 background were bred as Muc2^-/-^ x Muc2^+/-^ at the University of Gothenburg and offspring were genotyped [42]. C57BL/6 mice (Charles River Laboratories, Sulzfeld, Germany) were used as controls initially and were replaced with littermate Muc2^+/-^ mice as controls for the remainder of the study. Mice were kept under specific pathogen-free conditions and were provided with food and water ad libitum. Protocols were approved by the Gothenburg animal ethics committee (Göteborgs djurförsöksetiska nämnd; permits 310–2010 and 280–2012), and institutional animal use and care guidelines were followed. Mice were sacrificed by cervical dislocation after isoflurane anesthesia using an airstream of 2 l/min and 3.5% isoflurane. The general health of mice was monitored several times per week and animals were weighed once per week. Mice were sacrificed and an experiment was performed when one or more mice in a cage showed any sign of ill health such as rectal redness or swelling.

### Preparation of mouse tissues

Mice were sacrificed at 8–24 weeks of age. The colon was analyzed in all mice and the small intestine was analyzed in some mice. Single cell suspensions were prepared as described previously [43].

### Cytokine measurements

Concentrations of IL-2, IL-4, IL-6, IFN-γ, TNF, IL-17a and IL-10 in supernatants from saponine extracts of a 1 cm^2^ piece of mouse colon prepared as published previously [44] were determined using the mouse Th1/Th2/Th17 Cytokine kit (BD Biosciences, San Jose, CA) according to the manufacturer’s recommendations. The concentration of IL-1β and TGF-β were determined in the same supernatants by ELISA. IL-1β was measured using the Mouse IL-1β ELISA set (BD Biosciences, San Jose, CA) according to the manufacturer's protocol. To quantify activated TGF-β1, supernatants were acidified and neutralized prior to analysis with the Human/Mouse TGF-β1 ELISA (ebioscience, San Diego, CA) according to the manufacture's recommendation. Cytokine production in co-culture supernatants were quantified using Mouse Th17 6-Plex Bio-Plex Pro Assays (BIO-RAD, Hercules, CA) on a MagPix System (Luminex, Austin TX) according to the manufacturer’s protocol.

### Flow cytometry

Single cell suspensions of mouse LP cells were prepared and stained as published previously [45]. Cell viability was determined by staining with Live/Dead Fixable Aqua Dead Cell Stain (Life Technologies). The following monoclonal antibodies recognizing mouse molecules were used: anti- MHC-II-Alexa700, TCR-β-APC, CD19-APC, NK1.1-APC, CD11b-APC-Cy7, Ly6G-FITC, CD11c-Pacific Blue, CD103-PE, CD4-Alexa700, IL-17-PE-Cy7, IFN-γ-FITC, FoxP3-APC and CD64-APC. Intranuclear Foxp3, IL-17 or IFN-γ expression in CD4 T cells was evaluated using the Foxp3 staining set (eBioscience) following the manufacturer’s protocol.

Samples were acquired either with a LSR II flow cytometer (BD Biosciences) using DIVA software (BD Biosciences) or sorted with the FACSAriaIIμ cell sorter (BD Biosciences) to be used in subsequent proliferation assays. Purity of P1, P2, P3 or P4 myeloid cells was routinely 93–98%. Data was analyzed using FlowJo software (Tree Star, Ashland, OR).

### Proliferation assay

Single-cell suspensions of splenic lymphocytes from OT-II Ly5.1 mice were prepared and red blood cells were lysed in ammonium chloride buffer. Washed cells were enriched for CD4^+^ T cells by MACS cell sorting using mouse CD4^+^ T cell isolation Kit II (Miltenyi Biotec) and AutoMACS according to the manufacture's protocols to a purity of above 90%. The untouched CD4^+^ T cells were re-suspended at 5 x 10^7^/ml in PBS and incubated with 5 μM Cell Trace Violet (CTV; Life Technologies) at 37°C for 20 min. Cells were then blocked with FCS and washed, re-suspended and adjusted to 4.375 x 10^5^ cells/ml and kept on ice until further use.

Sorted LP DCs or MΦ were pulsed with Ova_323-339_ peptide (100 ng/ml) or ovalbumin (1μg/ml) (Sigma Aldrich) for 2 h at 37°C and extensively washed. They were then cultured at a ratio of 1:8 with 8.75 x 10^4^ CTV-labeled naive CD4^+^ OT-II cells at 37°C for 5 days. CTV dilution was assessed by flow cytometry.

### Quantitative real time PCR (qPCR)

Total RNA was extracted with the SV total RNA isolation system from Promega (Madison WI, USA) according to the manufacturer’s protocol. RNA quantity and purity were measured using a NanoDrop ND-1000 Spectrophotometer (NanoDrop Technologies, Inc. Wilmington DE, USA). RNA was reverse transcribed with the GoScript RT System (Promega) and qPCR was performed on a LightCycler480 thermal cycler (Roche Diagnostics, Basel Switzerland) using GoTaq qPCR master mix (Promega). Primers for CCL9, CXCL1 and CXCL2 were purchased as pre-tested and optimized PrimePCR Assays from Bio Rad (Hercules CA, USA). Primers for HPRT (fwd-5’ TCC TCC TCA GAC CGC TTT T 3’; rws-5’ CCT GGT TCA TCA TCG CTA ATC 3’) were designed using Primer3 software and purchased from Eurofins MWG Operon (Ebersberg, Germany). Specificity and efficiency was tested in initial analyses. Differential gene expression was determined using the 2^ΔΔCt^method normalizing to the Ct-value of HPRT.

### Statistical analysis

Statistical analyses were performed with GraphPad Prism 5.0 (GraphPad Software, La Jolla, USA). For the comparison of two independent groups the two-tailed nonparametric Mann-Whitney-U test was applied to assess statistical significance. One-Way ANOVA followed by Tukey’s multiple comparison test or Kruskal-Wallis test followed by Dunn’s multiple comparison was used for comparison between three or more groups. A p value below 0.05 was considered statistically significant. *p< 0.05, **p<0.01, ***p<0.001 indicate significance in the figures.

## Supporting Information

S1 Fig Neutrophil frequency in colon segments of color-coded mice. **(Figure A)** The frequency of neutrophils, identified as in Fig 1, in the respective colon segment of the same mice in Figs 3 and 4 is shown. **(Figure B)**. CXCL1, CXCL2 and CCL9 expression in colon segments of the different groups of Muc2^-/-^ mice. Total RNA was isolated from total cell suspensions from the same colon segments (proximal, middle or distal) from the same animals analyzed in Figs 3 and 4 and A. Five mice of each group were included in the qPCR analyses. Differential expression of the indicated genes was analyzed using the 2^ΔΔCT^ method with expression of HPRT as the endogenous reference gene. Symbols of the same color represent samples from the same colon segment of the same animal as in Figs 3 and 4. Statistical significance between groups was assessed using the Kruskal-Wallis test followed by Dunn’s multiple comparison test.
(TIF)

S2 Fig Similar populations of CD64^-^F4/80^-^Ly6C^-^ dendritic cell subsets are present in caudal lymph nodes, MLN and colon LP. Single cell suspensions of caudal lymph nodes, MLN and colon LP from Muc2^+/-^ mice were analyzed by flow cytometry. Representative dot plots from two independent experiments with three mice show the gating strategy used to identify mucosal DC subsets in the indicated tissues. Cells were gated as negative for Live/Dead Fixable Aqua Dead Cell Stain and then sequentially gated for the other markers, left to right, as shown in the figure.
(TIF)
