# Supplementary figures and images for: CD103+CD11b+ Dendritic Cells Induce Th17 T Cells in Muc2-Deficient Mice with Extensively Spread Colitis
Source: PLoS One. 2015 Jun 29;10(6):e0130750. doi: 10.1371/journal.pone.0130750 (PMC4487685; doi:10.1371/journal.pone.0130750)

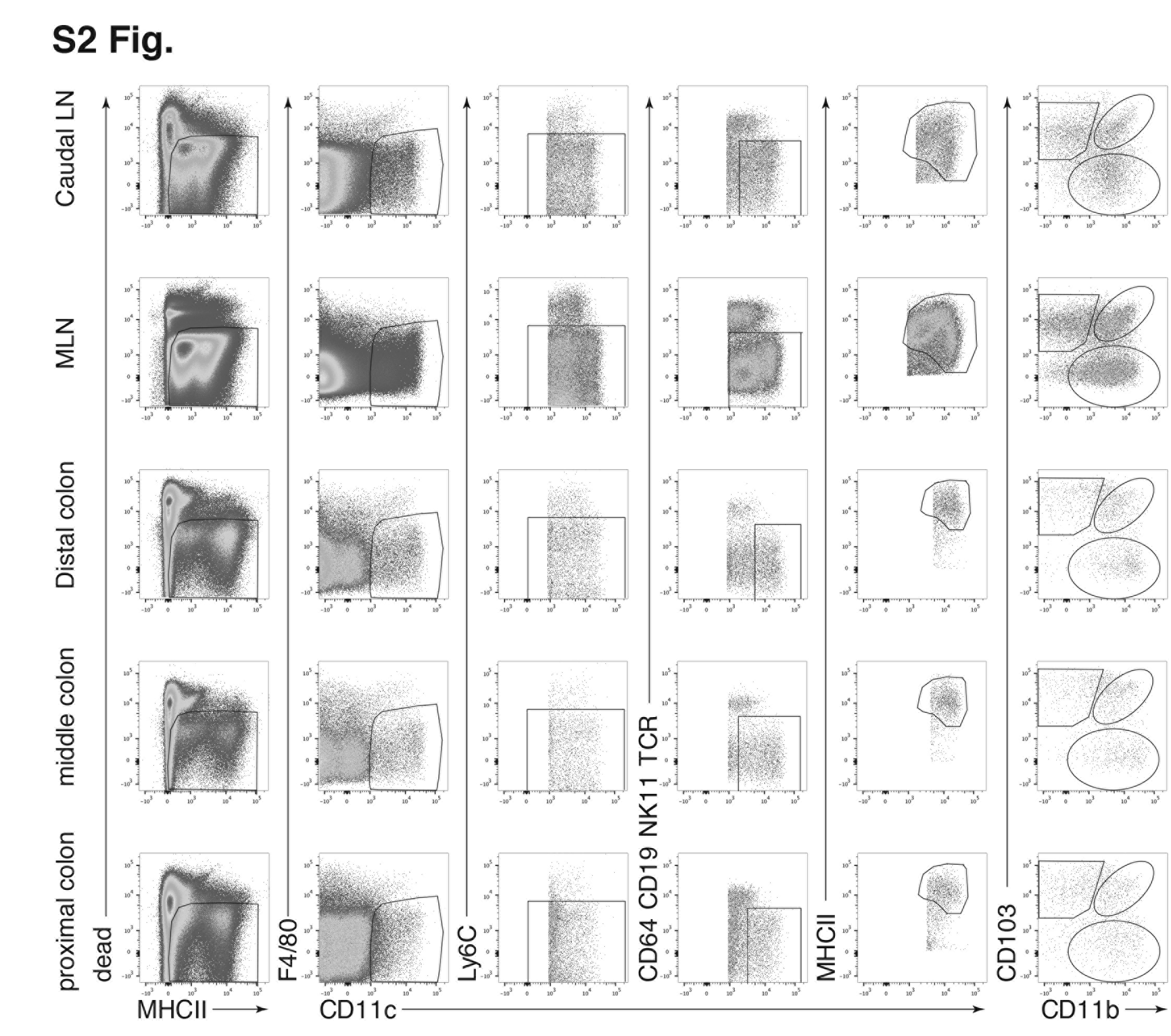

Supplement: S2 Fig — Single cell suspensions of caudal lymph nodes, MLN and colon LP from Muc2+/- mice were analyzed by flow cytometry. Representative dot plots from two independent experiments with three mice show the gating strategy used to identify mucosal DC subsets in the indicated tissues. Cells were gated as negative for Live/Dead Fixable Aqua Dead Cell Stain and then sequentially gated for the other markers, left to right, as shown in the figure. (TIF) [file pone.0130750.s002.tif]
